# In Vitro Assessment of the Effect of Antiepileptic Drugs on Expression and Function of ABC Transporters and Their Interactions with ABCC2

**DOI:** 10.3390/molecules22101484

**Published:** 2017-09-29

**Authors:** Gurpreet Kaur Grewal, Samiksha Kukal, Neha Kanojia, Krateeka Madan, Luciano Saso, Ritushree Kukreti

**Affiliations:** 1 Academy of Scientific and Innovative Research (AcSIR), CSIR-Institute of Genomics and Integrative Biology (CSIR-IGIB) Campus, Delhi 110007, India; gpkgrewal@gmail.com (G.K.G.); samkukal07@gmail.com (S.K.); kanojia29.2008@gmail.com (N.K.); 2 Genomics and Molecular Medicine Unit, Institute of Genomics and Integrative Biology (IGIB), Council of Scientific and Industrial Research (CSIR), Mall Road, Delhi 110007, India; krateekamadan.18@gmail.com; 3 Department of Physiology and Pharmacology “Vittorio Erspamer”, Sapienza University of Rome, P. le Aldo Moro 5, 00185 Rome, Italy; luciano.saso@uniroma1.it

**Keywords:** antiepileptic drugs, ABC transporters, ABCC2, efflux activity, ATPase assay

## Abstract

ABC transporters have a significant role in drug disposition and response and various studies have implicated their involvement in epilepsy pharmacoresistance. Since genetic studies till now are inconclusive, we thought of investigating the role of xenobiotics as transcriptional modulators of ABC transporters. Here, we investigated the effect of six antiepileptic drugs (AEDs) viz. phenytoin, carbamazepine, valproate, lamotrigine, topiramate and levetiracetam, on the expression and function of ABCB1, ABCC1, ABCC2 and ABCG2 in Caco2 and HepG2 cell lines through real time PCR, western blot and functional activity assays. Further, the interaction of AEDs with maximally induced ABCC2 was studied. Carbamazepine caused a significant induction in expression of ABCB1 and ABCC2 in HepG2 and Caco2 cells, both at the transcript and protein level, together with increased functional activity. Valproate caused a significant increase in the expression and functional activity of ABCB1 in HepG2 only. No significant effect of phenytoin, lamotrigine, topiramate and levetiracetam on the transporters under study was observed in either of the cell lines. We demonstrated the interaction of carbamazepine and valproate with ABCC2 with ATPase and 5,6-carboxyfluorescein inhibition assays. Thus, altered functionality of ABCB1 and ABCC2 can affect the disposition and bioavailability of administered drugs, interfering with AED therapy.

## 1. Introduction

Epilepsy is a neurological brain disorder, affecting 1–2% of the population worldwide [1]. Treatment involves the use of antiepileptic drugs (AEDs), however, about one-third of epilepsy patients fail to become seizure free [2]. Non-responsiveness to antiepileptic therapy is still an unsolved problem. The drug transporter hypothesis suggests that overexpression of ATP-binding cassette (ABC) transporters contribute to non-responsiveness. A study by Tishler et al. reported for the first time overexpression of ABCB1 in endothelial cells of epileptogenic brain tissue resected from patients with intractable epilepsy [3]. Since then overexpression of ABCB1 and other ABC transporters—such as ABCC1, ABCC2, ABCG2—has been reported by various groups in epileptogenic brain tissue of patients with refractory epilepsy [4,5,6,7] and in rodent as well as in mice models of temporal lobe epilepsies [4,5]. Several AEDs such as phenobarbital (PB), phenytoin (PHT), carbamazepine (CBZ) and valproate (VAL) seem to be inducers as well as substrates of the ABCB1 or ABCCs transporters and are likely to contribute to decreased brain concentrations of these drugs [5,8,9,10,11,12,13,14]. All these findings led to the formation of the multidrug-transporter hypothesis of resistance in epilepsy.

ABC transporters are the largest family of transmembrane transporters, with 48 human members. Out of these ABCB1, ABCC1/2 and ABCG2 play important roles in drug disposition and response [15]. ABCB1, ABCC2 and ABCG2 have apical localization [16], whereas ABCC1 is localized basolaterally [17]. These transporters, present at different tissue barriers, control the transport of a myriad of substrates. These four transporters play pivotal roles in the pharmaco- and toxicokinetics of xenobiotics as evident from their substrate specificities, tissue- and cell-specific expression patterns and experimental evidence [18].

Since most used AEDs are oral drugs, ABC transporters present in the intestine and liver are critical determinants of their efficacy before reaching the final target—the brain. Thus, it is important to understand the role of AEDs in influencing ABC transporters at these two major tissue barriers—intestine and liver—as they dictate the amount of drug entering systemic circulation [19]. It has been suggested previously that overexpression of ABCB1 in the brain alone does not explain the observed sub-therapeutic blood levels of AEDs. Overexpression of ABC transporters is a general phenomenon that involves overexpression at vascular endothelial cells and other peripheral tissues which reduces the oral bioavailability of drugs [20,21]. Increased expression of transporters in the intestine and liver can form a barrier that accelerates clearance of AEDs and thus reduces the clinical efficacy of oral medications [19,13,22]. Drug efflux pumps located at the canalicular membrane of hepatocytes actively transport the compounds into the bile canaliculus, helping to keep their intracellular concentration low [13].

Expression of ABCB1 in response to AEDs has been extensively studied in different cell lines [8,11,23,24]. Many studies reported induction of ABCB1 in response to first line AEDs while other reported no effect on expression [25,26,27]. There are fewer studies determining the effect of AEDs on other ABC transporters such as ABCC1, ABCC2, ABCG2 [11,13,22,28]. Moreover, the studies also lack data relating RNA and protein expression with functional transporter activity which demands further investigation. For instance, Lombardo et al. studied only the protein expression of ABCB1, ABCC1 and ABCC2 in immortalized rat brain microvascular endothelial cell lines, GPNT and RBE4, following exposure with the AEDs, topiramate (TOPI), PB, CBZ, tiagabine, levetiracetam (LEVI), and PHT [11]. Similarly, Oscarson et al. studied only RNA expression of drug metabolizing enzymes and drug transporters in the liver samples from epilepsy patients treated with CBZ [13]. The inducer drugs will clinically impact drug pharmacokinetics, further worsening the situation for non- responders, when such drugs are also substrates of ABC transporters. Lamotrigine (LAMO), oxacarbamazepine (OXCBZ), PB and PHT are definitely substrates of ABCB1 [29]. Evidences from various in vitro and in vivo studies indicate that CBZ is a substrate of ABCB1 and ABCC2 [29,30,31,32,33,34] while several other reports refute this [35,36,37,38,39]. Hence, the information regarding transport of AEDs by such transporters remains unclear. This study aimed to evaluate the effects of the first line AEDs such as PHT, CBZ, VAL and second line AEDs such as LAMO, TOPI, LEVI on expression and function of ABCB1, ABCC1, ABCC2 and ABCG2—major determinants of oral availability. Out of ABC transporters examined with AEDs, the maximal functional activity change was observed in ABCC2 in response to CBZ in both the cell lines under study. This observation made it important to discern the AEDs whose disposition can be affected by altered ABCC2 expression. Also, there are conflicting results for AEDs to be substrates of ABCC2 [31,37,40], so it is difficult to assess the clinical efficacy of which AEDs is affected by ABCC2. Thus, we identified the substrate relationship of AEDs with ABCC2, an emerging transporter of clinical importance recognized by the International Transporter Consortium [41,42,43]. To our knowledge, it is the first study examining the effects of six AEDs at three levels: mRNA, protein expression and functional activity in human colorectal adenocarcinoma (Caco2) and human liver carcinoma (HepG2) cells and evaluating the substrate relationship of AEDs with ABCC2 with two approaches—ATPase and efflux assays.

## 2. Results

### 2.1. Effects of AEDs on RNA Expression of ABC Transporters

After performing cell viability tests to determine non-cytotoxic doses, we examined the mRNA expression of ABCB1, ABCC1, ABCC2 and ABCG2 in Caco2 and HepG2 cells treated with therapeutic concentrations of PHT, CBZ, VAL, LAMO, TOPI and LEVI [44] (Figure S1). We have tabulated the list of AEDs under study, structure, mode of action, discovery and market details along with their enzyme inducing nature in Figure S2. Caco2 and HepG2 cell lines were used as in vitro system since intestinal and liver cells represent the major biotransformation sites of the body and known to express ABC transporters [45,46]. CYP3A4 was used as positive control gene as it is induced in the presence of PHT, CBZ, VAL and TOPI.

There was no change in expression of ABC transporters under study on treatment with PHT except for ABCB1 in HepG2 cells (Table 1). ABCB1 expression was also induced by more than 2-fold in response to treatment with another drug, CBZ in Caco2 and HepG2 (Table 1) cell lines. Similarly, expression of ABCC2 was significantly increased by more than 1.6-fold on treatment with CBZ in Caco2 and HepG2 cells (Table 1). In Caco2, ABCC1 expression was induced only at lower dose of 21 µM CBZ while the expression of both ABCC1 and ABCG2 was induced only at higher dose of 42 µM CBZ in HepG2. Cell line specific response was observed with VAL treatment on ABCB1 expression, with a 2-fold decrease at 600 µM in Caco2 and 3-fold induction at 600 µM in HepG2. Out of second line AEDs only TOPI induced expression of ABCB1, ABCC1, ABCC2 and ABCG2 in cell lines under study (Table 1).

### 2.2. Effects of AEDs on Protein Expression and Functional Activity of ABC Transporters

**Figure 1:**
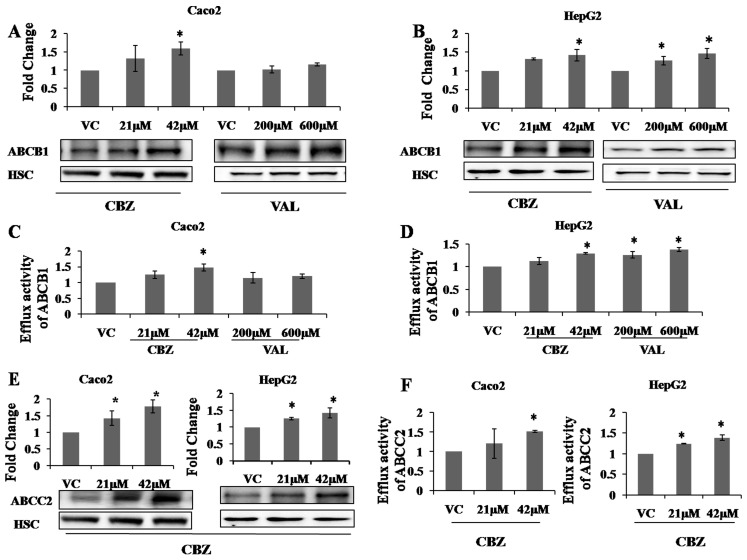
Effect of AEDs (carbamazepine and valproate) on protein expression and functional activity of ABCB1 and ABCC2 in Caco2 and HepG2 cells. (**A**,**B**,**E**) Immunoblot analysis of whole cell lysates treated either carbamazepine (CBZ) or valproate (VAL) for 72 h. HSC was used to normalise the results. Data shown are means ± S.D. from atleast (*n* = 3) independent experiments. Statistical significance was determined using ANOVA and Tukey’s HSD post hoc test * *p* < 0.05, compared with vehicle control (VC); (**C**,**D**,**F**) Efflux activity of ABCB1 and ABCC2 transporter was measured using specific fluorescent substrates (ABCB1: rhodamine; ABCC2: carboxyfluorescein diacetate) after treatment with either CBZ or VAL for 72 h as indicated. Accumulation of fluorescent substrate in cells was measured in the presence or absence of inhibitor (ABCB1: verapamil (50 µM); ABCC2: MK571 (100 µM)). Fluorescence intensities were normalized with total protein content. Data are expressed as mean ± S.E.M. (*n* = 3) and statistical significance was determined using ANOVA and Tukey’s HSD post hoc test * *p* < 0.05.

After studying the changes in mRNA expression of ABCB1, ABCC1/2 and ABCG2, we studied the effect on protein expression. Here, we discussed and showed data of only those AEDs for which we observed significant changes in protein expression of transporters. Changes in protein expression of ABCB1 were studied with CBZ and VAL, whereas those of ABCC2 with CBZ were studied, after 72 h treatment with the respective drugs. Proteins were deglycosylated with PNGase F during sample preparation to get distinct protein bands in the blots. As observed for mRNA, a similar trend was observed for protein expression of ABCB1 in response to CBZ treatment. Expression of ABCB1 was induced in the presence of CBZ in both Caco2 (1.59 ± 0.18 for 42 µM CBZ) (Figure 1A) and HepG2 (1.42 ± 0.15 for 42 µM CBZ) (Figure 1B) cell lines. VAL had no significant effect on ABCB1 protein expression in Caco2 (Figure 1A), while it induced the same (1.27 ± 0.11 for 200 µM VAL; 1.46 ± 0.13 for 600 µM VAL) in HepG2 cells (Figure 1B). Further, expression of ABCC2 was determined in response to CBZ treatment and significant increase in ABCC2 protein expression was observed in both cell lines (Caco2: 1.42 ± 0.21 for 21 µM CBZ; 1.77 ± 0.19 for 42 µM CBZ) (HepG2: 1.25 ± 0.03 for 21 µM CBZ; 1.42 ± 0.14 for 42 µM CBZ) (Figure 1E).

Further, efflux activity of ABCB1 was determined after treatment of CBZ and VAL using rhodamine 123, a known substrate of ABCB1. Verapamil was used as an inhibitor of ABCB1. CBZ significantly increased the efflux activity of ABCB1 in both cell lines (Caco2: 1.47 ± 0.10 for 42 µM CBZ) (HepG2: 1.29 ± 0.01 for 42 µM CBZ) (Figure 1C,D). VAL had a differential effect on two cell lines under study, with no significant change in Caco2 and increased efflux activity in HepG2 cells (HepG2: 1.25 ± 0.07 for 200 µM VAL; 1.37 ± 0.04 for 600 µM VAL) (Figure 1C,D). The efflux activity of ABCC2 was determined after CBZ treatment using 5,6-carboxyfluorescein diacetate (CFDA), a known substrate of ABCC2.

MK571 was used as an inhibitor for ABCC2. ABCC1 and ABCC2 show the same substrate specificities and both have been found to transport 5,6-carboxyfluorescein (CF) [47,48,49,50], but we observed overexpression of ABCC2 and not ABCC1 in response to CBZ, so the figure (Figure 1F) represents the efflux of ABCC2. A significant increase in efflux activity of ABCC2 was observed after treatment with CBZ in Caco2 (1.52 ± 0.02 for 42 µM CBZ) and HepG2 (1.24 ± 0.01 for 21 µM CBZ; 1.38 ± 0.06 for 42 µM CBZ) (Figure 1F). Thus, efflux activity data were consistent with protein expression data.

### 2.3. Effects of AEDs on ABCC2 ATPase Function

**Figure 2:**
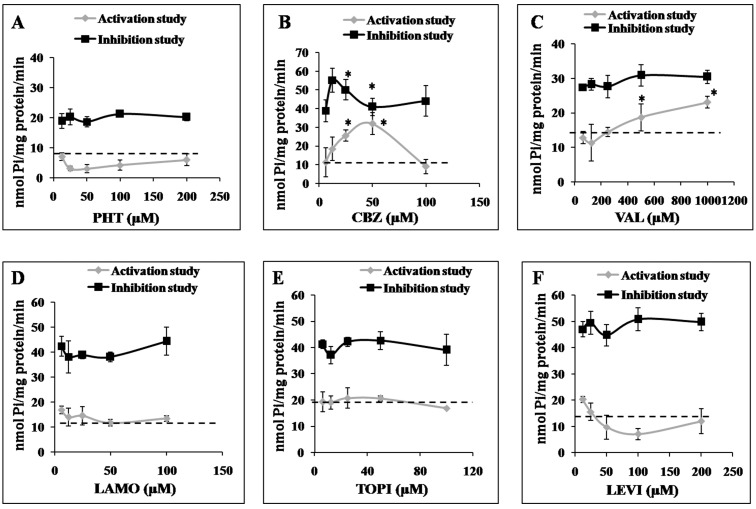
Increasing concentrations of AEDs: phenytoin (PHT) (**A**), carbamazepine (CBZ) (**B**), valproate (VAL) (**C**), lamotrigine (LAMO) (**D**), topiramate (TOPI) (**E**) and levetiracetam (LEVI) (**F**) incubated with vesicles expressing ABCC2. Results are expressed as the amount of produced Pi per milligram of total membrane protein per min. Data are presented as means ± S.D obtained from three independent experiments. Statistically significant differences between stimulated control and in inhibition assays (* *p* < 0.05) were determined using unpaired *t* tests.

The interactions of AEDs (substrate interactions) were undertaken for ABCC2 and not for ABCB1 as there are extensive studies for ABCB1 identifying a substrate relationship of AEDs with ABCB1. The ABCC2 transporter efflux activity is associated with ATP hydrolysis and is modulated in the presence of substrates or inhibitors. To characterize the interactions of AEDs with ABCC2, the effect of a range of different concentrations of AEDs on vanadate-sensitive ATPase activity in isolated insect Sf9 cell membranes overexpressing human ABCC2 was determined. In the activation assay, increasing concentration of CBZ and VAL (not the PHT, LEVI, LAMO) stimulated ATPase activity from the baseline measurement (Figure 2B,C). CBZ reduced ATPase activity at the higher dose. TOPI only slightly increased the ATPase activity (Figure 2E). The inhibition assay was done in the presence of test drug and known activator of ABCC2-sulfasalazine, which maximally stimulates the ATPase activity. Only CBZ reduced the stimulated vanadate sensitive ATPase activity in the inhibition assay (Figure 2B).

### 2.4. Inhibition of 5,6-Carboxyfluorescein Efflux (CF) via ABCC2 by AEDs

**Figure 3:**
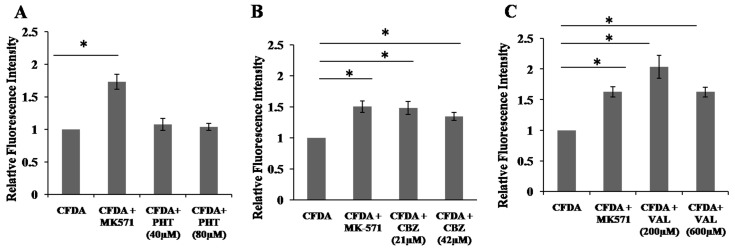

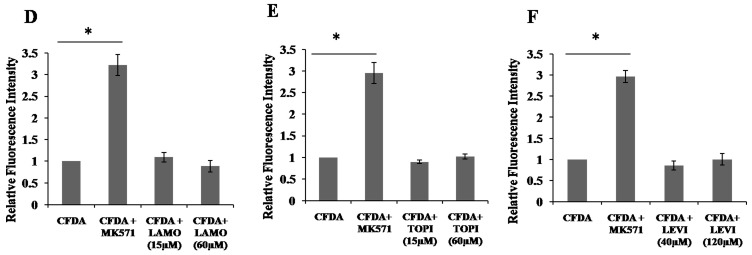
Efflux assay to study substrate relationship of AEDs using a fluorescent substrate, carboxyfluorescein diacetate. (**A**–**F**) Cells were treated with either AED indicated or inhibitor of ABCC2 (MK-571). Carboxyfluorescein diacetate (substrate of ABCC2) accumulation in cells was measured in the presence or absence of MK571 (100 µM). CFDA represents vehicle control (VC). Results are represented as mean fluorescent intensity normalized with total protein concentration. Data is expressed as mean ± S.E.M. (*n* = 3) and statistical significance is determined using ANOVA and Tukey’s HSD post hoc test. * *p* < 0.05, VC (vehicle control, 0.1% DMSO) vs. CBZ/VAL or VC vs. MK-571.

The AED transporting activity of ABCC2 was further measured by estimating the AEDs induced inhibition of ABCC2 mediated CF transport in HepG2 cells. The membrane permeable CFDA is converted into membrane impermeable CF in the cytosol by intracellular esterases, which can then move out from the cell only through transporter. Positive control MK-571 reduced the transport of the fluorescent molecule CF and resulted in accumulation of CF inside cells, indicating inhibition of transport of CF. Results were represented as mean fluorescent intensity normalized with total protein concentration. PHT, LAMO, TOPI and LEVI did not inhibit the CF efflux. However, CBZ and VAL clearly increased CF fluorescent intensity compared to vehicle control and thus, inhibiting CF efflux (Figure 3).

## 3. Discussion

ABC transporters play a significant role in drug disposition and any change in their expression and activity can have profound influence on pharmacological response. Extensive clinical and experimental studies substantiate the role of ABC transporters in pharmacoresistant epilepsy [3,11,24,51,52,53,54,55]. Moreover, genetic studies worldwide have tried to associate polymorphisms of ABC transporters to the AED response but data seems to be inconclusive. The study by Siddiqui et al. was the first to report positive association between C3435T polymorphism of ABCB1 and resistance to AEDs [56]. Since, then several groups have attempted to replicate the findings across different populations but the results were non-reproducible and inconclusive [57,58]. A study by our group also failed to find any positive association between ABCB1 polymorphisms (C1236T, G2677T/A and C3435T) and drug response in epilepsy patients [59]. Similarly, studies investigating the role of ABCC2 variants were also inconsistent [60,61]. A study by Nguyen et al. which examined the functional significance of promoter polymorphisms of ABCC2, concluded that the studied ABCC2 promoter SNPs are not critical for its transcriptional regulation and possibly other transcriptional modulators may be controlling variability in expression and function of ABCC2 [62]. Thus, we investigated the effect of, xenobiotics (AEDs themselves), as the regulators of ABC transporter expression. The present study focused on the effect of first (PHT, CBZ, VAL) and second line (LAMO, TOPI, LEVI) AEDs on expression and functional activity of ABC transporters ABCB1, ABCC1, ABCC2 and ABCG2. To understand the effect of AEDs, we chose two cell lines viz. Caco2 and HepG2 representing two important tissue barriers viz. intestine and liver, which can affect bioavailability of drug at the initial site of drug absorption. The flow of the strategy used in our study is summarized in Figure S3.

A differential effect of first and second line AEDs was observed on ABCB1, ABCC1, ABCC2 and ABCG2 in two cell lines under study. A dose-dependent induction effect of CBZ was observed in the expression of ABCB1 and ABCC2 at RNA and protein levels in both the cell lines. In agreement with this, CBZ also significantly increased the functional activity of ABCB1 and ABCC2 as shown from the rhodamine and carboxyfluorescein substrate assays, respectively. Previously also various groups investigated the effect of CBZ on ABCB1 and ABCC2 in different model systems. For instance, Owen et al. examined the effect of CBZ in lymphocytes and documented elevated levels of ABCB1 at both RNA and protein level [63]. A study conducted to assess the effect of CBZ on ABCB1 and ABCC2 in human duodenal samples reported increase in mRNA expression of both ABCB1 and ABCC2 on CBZ administration to healthy subjects. ABCC2 protein expression was also induced, but ABCB1 protein expression was unaffected [8]. Wen et al., performed a study to evaluate the effect of chronic administration (21 days) of CBZ on ABCB1 in male Sprague–Dawley rats and reported induction of ABCB1 expression and function [24].

VAL treatment displayed cell line specific regulation of the ABCB1 transporter. It significantly reduced the RNA expression of ABCB1 with no change at protein and functional level in Caco2, while in HepG2 it induced the expression of ABCB1 at RNA, protein and functional level. A similar disparity in studies can also be found in the literature when VAL was administered in different cell models. For example, VAL was shown to increase ABCB1 expression and function in human colon adenocarcinoma cell line SW620 and acute myelogenous leukaemia KG1a cells [64]. Along similar lines, using transfection reporter assay and real-time RT-PCR, Cerveny et al. reported that VAL can potentially induce the expression of ABCB1 in cells transfected with expression vectors encoding constitutive androstane receptor or pregnane X receptor [65], whereas, a study by Weiss et al. found inhibition of ABCB1 in a MDR1-transfected kidney cell line (LLC) at therapeutic concentrations (250–500 μM) of VAL [30]. Another study using rhodamine 123 efflux assay also found inhibition of ABCB1 functional activity by VAL in human brain endothelial cell line hCMEC/D3 at 300 µM therapeutic concentration of VAL, but the effect was lost at higher concentrations. It is being suggested that depending on the cell line and drug concentration, VAL can both induce and inhibit ABCB1 functionality [66]. Thus, biphasic effects of VAL observed on ABCB1 could be attributed to differential interaction of VAL with specific transcriptional factors in cell lines investigated at specific concentrations. This differential effect merits further mechanistic investigation.

PHT only induced mRNA expression of ABCB1 in HepG2 cells but no significant change in protein expression was observed (data not shown). To the contrary, earlier study reported modest induction of protein expression of ABCB1 by PHT treatment (1.3 ± 0.1 fold), which was conducted in human colon adenocarcinoma, LS1 80/WI, and its adriamycin-resistant subline, LS1 80/AD5O to determine the effect of range of xenobiotics on expression of ABCB1 [23]. Another study, performed immunohistochemistry in endothelium and parenchyma of several brain regions of female Wistar rats to assess the effect of chronic administration of PHT and reported no significant increase in ABCB1 expression, which was in agreement with our observation [27].

Among the second line AEDs, although TOPI showed an increase in mRNA levels of ABCB1 and ABCG2 under study, none of these AEDs had any significant effect on protein and functional levels (data not shown). The fact that changes observed in transcript expression are not always translatable is highlighted by finding of Rubinchik Stern et al. [28]. This could be due to number of regulations operating at different levels of gene expression. The control can be at the point of RNA processing, RNA stability (post-transcriptional modifications), protein synthesis and degradation [67]. The observation of second line AEDs, of not having inducing effect on transporters under study can be correlated from the literature, where in general, LAMO, TOPI and LEVI are considered as a non-enzyme inducing AEDs [44].

Thus, ours is the first in vitro study examining the effect of six AEDs on four ABC transporters (ABCB1, ABCC1, ABCC2 and ABCG2) at the RNA, protein and functional levels in two cell models (Caco2 and HepG2). CBZ caused significant induction in expression of ABCB1 and ABCC2 in HepG2 and Caco2 cells whereas VAL caused significant induction in expression and function of ABCB1 in HepG2. It is important to understand the mechanism of regulation of ABC transporters in response to AEDs as this would help in identifying targets that can be modulated to deal with the problem of non-responsiveness in a better way. In our recently published article, we explored the molecular mechanism of CBZ induced ABCC2 expression and focused on CBZ-pregnane X receptor (PXR) interaction. By using nuclear translocation and RNA interference experiments, we confirmed the direct involvement of PXR in CBZ induced ABCC2 expression. Further, our in silico comparative structural analysis suggested the agonist type of mode of interaction of CBZ with PXR and role of Gln285 residue of PXR in CBZ-PXR interaction was deciphered [68].

Notably, the change in the functional activity of both ABCB1 and ABCC2 was significantly induced only in response to CBZ treatment in both HepG2 and Caco2, with slightly higher change observed for ABCC2. ABCC2 being the less explored transporter compared to ABCB1 [69] was further examined for its interaction with AEDs. The interaction of drug as a substrate or an inhibitor of the transporter can either lead to subtherapeutic or toxic concentrations, thus interfering with the therapy. Cell-based efflux assays in the presence of CFDA efflux indicated CBZ and VAL to be the possible substrates of ABCC2 as revealed by increased CF fluorescence inside the cells. This observation was further strengthened by ATPase assay. The measurement of ATPase activity revealed that CBZ and VAL stimulated ABCC2 ATPase activity. Activation of the ATPase activity of a transporter by the tested compound indicates about being a quickly transported substrate [70]. We further observed that CBZ slightly decreased the sulfasalazine activated ATPase activity of ABCC2. Previously, few studies have shown positive evidences for CBZ being the substrate of ABCC2. Kim et al. found CBZ to be a substrate of ABCC2 using ATPase assay and FACScan flow cytometer [31]. The involvement of ABCC2 in regulation of extracellular brain concentrations of CBZ was also confirmed by in vivo microdialysis by Potschka et al. in rats in the presence of inhibitor probenecid [32]. On the contrary, a study showed CBZ not to be transported by ABCC2 using concentration equilibrium transport assay (CETA) in polarized kidney cell lines (LLC, MDCKII) transfected with human ABCC2 [38]. Similarly, Radisch et al. found no substrate relationship of CBZ with ABCC2 using in vitro approach [37]. Congruent to our finding, several studies demonstrated the interaction of VAL with MRP and indicated substrate relationship [9,71]. Bachmeier and Miller (2005) indicated substrate relationship of VAL with ABCB1 and MRP by showing increased accumulation of the mixed ABCB1 and MRP probe, 2′,7′-bis(2-carboxyethyl)-5(6)-carboxyfluorescein in bovine brain microvessel endothelial cell (BBMEC) monolayers [72]. However, the study by another group failed to show the transport of VAL by ABCC2 [73]. Further in this study, we did not observe any interaction of PHT, LAMO, TOPI and LEVI with ABCC2 which is in line with the literature [38]. Hence, by using two in vitro assays, we identified CBZ and VAL as possible substrates of ABCC2 and negated any interaction of other four AEDS (PHT, LAMO, TOPI and LEVI) with ABCC2. This way we have extended and validated the earlier findings in different model systems, although, comprehensive understanding of exact substrate/inhibitor nature of test compound is still required by adopting a more direct approach, where efflux of AEDs can be examined in response to treatment with AEDs themselves. There is a need for further in vivo studies, including positron emission tomography (PET) with labelled MRP substrates with appropriate controls.

Thus, the increased functionality of ABCC2 transporter and substrate interaction with CBZ and VAL was observed by in vitro approach in Caco2 and HepG2 cells which may reduce their concentrations at final disposition site-brain. The peripheral overexpression of ABC transporter can be developed as an objective marker of drug resistant epilepsy and this could help in drug dose adjustments in patients [21] as demonstrated by previous study where ABCB1 overexpression was used as guide for clinical treatment [74].

There are ample evidences where overexpression of ABCC2 was observed in epilepsy patients [13,51,52], but the specific contributions of AEDs in the transporter overexpression are still not clear. Further, lack of concrete evidence about transport of AEDs by efflux transporters at therapeutic concentrations is still the weakest connection of transporter hypothesis [75]. There are both supporting and contradictory in vitro studies exploring the substrate relationship of CBZ with ABCC2 [31,37]. Though positive substrate relationship evidences for VAL from in vitro studies and for CBZ from in vivo studies are available [9,32], there is need of clinical evidence that support ABC transporter-mediated AED transport in the human brain. Using PET and magnetic resonance imaging, increased ABCB1 transport activity has been demonstrated in patients with drug-resistant epilepsy [76,77,78]. In order to examine the contribution of overexpression of ABC transporters (in response to AEDs) to pharmacoresistance, there is need of PET imaging in AED responsive and resistant patients using labelled ABC transporter substrates.

One limitation of our study is that we have administered single doses of AEDs under study, and it needs to be determined how chronic exposure of AEDs affects the transporter expression in vitro and in vivo. Though there are studies examining the effect of AEDs in vivo but still not reached definite conclusion. For example, a study investigated the effect of chronic administration (21 days) of three drugs namely PB, PHT and CBZ on ABCB1 in male Sprague–Dawley rats and observed induction of ABCB1 expression and function [24]. Similarly, another study found no significant increase in ABCB1 protein expression after prolonged treatment of 11 days with PB or PHT in brain tissue of rats [27]. To the contrast, Oscarson et al. reported upregulation of mRNA expression of ABCC2 and ABCG2 in liver samples of patients with epilepsy treated with CBZ [13]. Thus, there is need of more functionally relevant studies to understand pharmacoresistance, where the transporter expression is related with reduced penetration of AEDs and leading to poor drug response.

## 4. Materials and Methods

### 4.1. Cell Culture

Caco2 and HepG2 cells were procured from National Centre for Cell Science (Pune, India). Both the cell lines were authenticated by CellSure:Human Cell line Authentication Service (Lifecode, New Delhi, India) and were used for experiments at passage number below 45. Cells were cultured in Dulbecco’s modified Eagle’s medium high glucose media (DMEM, Sigma-Aldrich, St. Louis, MO, USA) supplemented with 100 units/mL penicillin, 100 μg/mL streptomycin. HepG2 medium was supplemented with 10% fetal bovine serum (Invitrogen, Carlsbad, CA, USA). For Caco2, medium was supplemented with 0.1 mM nonessential amino acids, 20% fetal bovine serum. Cells were maintained at 37 °C in humidified 5% CO_2_ incubator.

### 4.2. Drug Treatments

Drugs PHT, CBZ, VAL, LAMO, TOPI, and LEVI were purchased from Sigma-Aldrich and dissolved in dimethyl sulphoxide (DMSO), except for LEVI which was dissolved in water. Preconfluent cells were treated with drugs in therapeutic range [44].

### 4.3. Cell Viability Test

Caco2 and HepG2 cells were seeded at 10,000 cells per well in 96-well plates and incubated at 37 °C overnight. Cells were then treated with various doses of drugs under study for 72 h, after which the 3-(4,5-dimethylthiazol-2-yl)-2,5-diphenyltetrazolium bromide reagent (MTT) (5 mg/mL; Ameresco, Fountain Parkway, Solon, OH, USA) was added and the assay was performed as described earlier [79].

### 4.4. RNA Extraction and Real Time RT-PCR

Caco2 (1.5 × 10^6^ cells/well) and HepG2 (2 × 10^6^ cells/well) cells were seeded in 6-well plate. On reaching 60% confluency, cells were treated with therapeutic range of drugs followed by RNA isolation after 24 h using TRIzol (Invitrogen) method according to manufacturer’s protocol and quantified through 260/280 absorbance. One μg of total RNA was reverse transcribed using random hexamers. The cDNA was subjected to quantitative RT-PCR using SYBR Green RT-PCR Master Mix (Applied Biosystems, Foster City, CA, USA) and gene-specific primers (Table 2) according to the manufacturer’s instructions (StepOne Plus RT-PCR system; Applied Biosystems). Data were analyzed using the Pfaffl method [80].

### 4.5. Western Blots

Caco2 (0.5 × 10^6^ cells/well) and HepG2 (1 × 10^6^ cells/well) cells were seeded in 6-well plate one day prior to the treatment. 72 h after drug treatment, total protein lysates were prepared using RIPA lysis buffer (50 mM Tris–HCl, pH 8, 150 mM NaCl, 1% NP-40, 0.5% sodium deoxycholate, 0.1% SDS) and quantified by the BCA protein estimation kit (Thermo Fischer Scientific, Scoresby, Australia). Protein samples were treated with PNGase F (New England Biolabs, Ipswich, MA, USA) to deglycosylate proteins and to get discrete single bands. Equal amount of protein extracts (50 µg) were electrophoresed on 6% and 10% SDS-PAGE gel. On transfer to nitrocellulose membrane, the membranes were blocked with 5% bovine serum albumin in Tris buffer saline (20 mM Tris, 150 mM NaCl, pH 7.4) with 0.1% Tween-20 for 1h, and incubated overnight at 4°C with anti-ABCB1 (MDR1/ABCB1 (E1Y7S) Rabbit mAb #13978), anti-ABCC1(MRP1/ABCC1 (D7O8N) Rabbit mAb #14685), anti-ABCG2 (Rabbit mAb #4477) (Cell Signaling Technology, Danvers, MA, USA) , anti-ABCC2 (ab3373 mouse mAb), anti-Vinculin (sc-73614 mouse mAb) (Abcam, Cambridge, MA, USA) and anti-HSC70 (B-6) (sc-7298 mouse mAb) (Santa Cruz Biotechnology, Santa Cruz, CA, USA) antibodies. After incubation with the secondary anti-mouse HRP-conjugated antibody, the bands were visualized using ECL detection reagents (Pierce Biotechnology, Rockford, IL, USA) on Chemi Doc™ MP Imaging system (Bio-Rad, Hemel Hempstead, UK) and quantified with the AlphaImager 3400 software (Alpha InnoTech Corp., San Leandro, CA, USA).

### 4.6. Functional Activity Assay

Caco2 (0.1 × 10^5^ cells/well) and HepG2 cells (0.5 × 10^5^ cells/well) were seeded in a 12-well plates one day prior to the drug treatment. Cells were treated with either DMSO or AEDs under study for 72 h. The medium used for the functional activity assay was Opti-MEM as it is commonly used medium for functional activity assays due to its reduced serum concentrations that does not interfere with fluorescence [46]. Media and 1×PBS buffer used was at pH of 7.4.

#### 4.6.1. Functional Activity Using Rhodamine

After the drug treatment, cells were washed with PBS and Opti-MEM media containing rhodamine123 (5 µM), a substrate of ABCB1 was added for 15 min at 37 °C in 5% CO_2_. Media was discarded and wash was given with ice cold PBS thrice, followed by incubation for 15 min at 37 °C in 5% CO_2_ in the Opti-MEM medium, with or without ABCB1 inhibitor, verapamil (5 µM) (Sigma-Aldrich) to allow efflux to occur. Efflux was stopped by washing with ice-cold PBS thrice and cells were lysed with a solution of 1% Triton X100, kept at 37 °C for an additional 15 min before fluorescence was measured and fluorescence readings were obtained using Infinite 200 PRO multimode plate reader (Tecan, Männedorf, Zurich, Switzerland) with excitation and emission wavelengths of 485 and 538 nm, respectively.

#### 4.6.2. Functional Activity Using Carboxyfluorescein Diacetate

After the drug treatment, cells were washed with PBS and Opti-MEM medium, with or without MK-571 (100 µM) inhibitor of ABCC2 was added for 15 min at 37 °C in 5% CO_2_. Thereafter, Opti-MEM media having CFDA (5 µM, Sigma-Aldrich) was added for 30 min at 37 °C in 5% CO_2_ in the presence and absence of MK571 (100 µM). After incubation, CFDA was washed out with ice-cold PBS thrice and incubated for 30 min at 37 °C in 5% CO_2_ to allow efflux to occur. Efflux was stopped by washing with ice-cold PBS and cells were lysed with a solution of 1% Triton X100, kept at 37 °C for an additional 15 min before fluorescence was measured and fluorescence readings were obtained using Infinite 200 PRO multimode plate reader (Tecan) with excitation and emission wavelengths of 492 and 518 nm, respectively.

#### 4.6.3. Data Analysis

Functional activity was expressed as efflux activity and was calculated using the formula: % Efflux activity = 100 × ((FI_inh_ − FI_0_)/FI_inh_), wherein FI_inh_ and FI_0_ are the fluorescence intensity values measured in the presence and absence of inhibitor [81]. Fluorescence intensities were normalized with total protein content.

### 4.7. ATPase Assay

An MRP2 ATPase kit (Solvo Biotechnology, Budaors, Hungary) was used to evaluate the substrate relationship of AEDs with MRP2. The assay was conducted according to the manufacturer’s instructions. Membrane preparations with overexpressed MRP2 transporter was used in study and specific ABC transporter related ATPase activity was measured as the vanadate-sensitive portion of total ATPase activity that is modulated by interacting compounds was measured. In the activation assay which was conducted only in presence of test drug, substrates transported may increase baseline vanadate sensitive ATPase activity while in the inhibition assay; reductions in the activity associated with the presence of a known activator of the transporter (sulfasalazine-reference compound) and test drug were measured. The results were expressed as vanadate-sensitive ATPase activities.

### 4.8. Efflux Assay Using Fluorescent Substrate-5,6-Carboxyfluorescein Efflux (CF)

An efflux assay using the fluorescent substrate CFDA was used to understand substrate or interactions of AEDs with ABCC2 in one of cell models used in the study. Both cell lines (HepG2 and Caco2) have functional ABCC2 transporters as demonstrated during functional activity assays, so either of them can be used for substrate studies. For the substrate study, inhibition of CF via ABCC2 by AEDs was studied in one of cell models (HepG2) having expression of ABCC2. This assay was conducted as mentioned in Section 4.6.2, but the drug to be tested was added at the time of efflux assay when inhibitor of ABCC2 was added. The results were represented as mean fluorescent intensity normalized with total protein concentration.

### 4.9. Statistical Analysis

The results were represented as mean ± standard deviation of at least three or more as indicated independent experiments. To determine statistical significance, either Student’s *t* test or One-way ANOVA followed by Tukey HSD post-hoc test was performed as appropriate. Probability values were considered significant at a *p* < 0.05.

## 5. Conclusions

Here, in this study we tried to fill in lacuna in determining the effect of six AEDs on four important ABC transporters by studying changes at RNA and protein expression and relating them to the functional activity of transporters which is largely lacking in the literature. In our study, we demonstrated for the first time the changes in Caco2 and HepG2 cell models in RNA, protein expression and functional activity of ABCB1 and ABCC2 in response to CBZ treatment and of ABCB1 in response to VAL treatment. It can be concluded from our study that CBZ causes a significant induction in expression of ABCB1 and ABCC2 in Caco2 and HepG2 cells. The functional activity was also significantly elevated for ABCB1 and ABCC2 (Caco2: ABCC2:1.52 fold vs. ABCB1:1.48 fold) (HepG2: ABCC2:1.38 fold vs. ABCB1:1.29) in response to CBZ treatment. VAL causes significant induction in expression and function of ABCB1 in HepG2. Therefore, therapeutic concentrations of these drugs could contribute to the increased functionality of transporters. ABCC2, being a less explored transporter, was further examined for the substrate relationships of six AEDs in different model systems or different approaches than previous findings [31,38]. We demonstrated the interaction of CBZ and VAL with this transporter using in vitro assays. We have tried to solve inconclusiveness in substrate relationship of CBZ with ABCC2 by using insect cells membrane vesicle preparation expressing ABCC2 for ATPase assay and HepG2 cells for CF efflux assay, which was different from the previous reported studies [31,37]. This way we have extended and strengthened the earlier findings. Thus, it can be inferred from our study that CBZ and VAL could modulate the disposition and bioavailability of co-administered drugs or endobiotics, which are substrates of ABCC2. This necessitates the need to explore the molecular mechanism of transporter regulation in response to these drugs that would aid in improving therapeutic outcome. Our recent study was a step toward the understanding of molecular mechanism where role of PXR and its residue Gln285 in CBZ induced ABCC2 expression was demonstrated [68]. Our lab is currently extending this work to understand the regulation at the final target site that is blood brain barrier.

## Figures and Tables

**Table 1:** Effect of antiepileptic drugs (AEDs) on mRNA expression of ABCB1, ABCC1, ABCC2 and ABCG2 in Caco2 and HepG2 cells.

DRUG	DRUG Concentration	Caco2	HepG2	Caco2	HepG2	Caco2	HepG2	Caco2	HepG2	Caco2	HepG2
		ABCB1		ABCC1		ABCC2		ABCG2		CYP3A4	
Phenytoin (PHT)	40 µM	1.09 ± 0.22	1.23 ± 0.15 *	1.17 ± 0.36	1.02 ± 0.12	1.09 ± 0.28	0.91 ± 0.25	1.19 ± 0.28	0.98 ± 0.31	1.25 ± 0.01 *	1.34 ± 0.01 *
	80 µM	1.02 ± 0.21	1.39 ± 0.31 *	1.01 ± 0.39	0.69 ± 0.01	1.04 ± 0.34	1.00 ± 0.19	1.17 ± 0.22	1.08 ± 0.14	1.28 ± 0.01 *	2.21 ± 0.26 *
Carbamazepine (CBZ)	21 µM	2.35 ± 0.07 *	1.69 ± 0.46 *	1.44 ± 0.37 *	1.12 ± 0.33	1.64 ± 0.15 *	1.68 ± 0.16 *	0.84 ± 0.23	0.95 ± 0.28	2.55 ± 0.08 *	1.49 ± 0.09 *
	42 µM	2.27 ± 0.01 *	2.26 ± 0.33 *	1.19 ± 0.12	1.57 ± 0.17 *	1.73 ± 0.23 *	1.74 ± 0.12 *	0.81 ± 0.16	1.37 ± 0.09 *	3.30 ± 0.15 *	2.83 ± 0.25 *
Valproate (VAL)	200 µM	0.64 ± 0.22 *	2.23 ± 0.21 *	1.15 ± 0.22	1.10 ± 0.27	0.62 ± 0.24	0.55 ± 0.19	1.13 ± 0.35	1.05 ± 0.08	2.11 ± 0.04 *	2.68 ± 0.13 *
	600 µM	0.55 ± 0.14 *	3.04 ± 0.27 *	1.15 ± 0.20	1.33 ± 0.64	0.83 ± 0.04	0.66 ± 0.11	1.07 ± 0.37	1.41 ± 0.26 *	3.16 ± 0.16 *	3.52 ± 0.27 *
Lamotrigine (LAMO)	15 µM	1.24 ± 0.13	1.06 ± 0.01	1.17 ± 0.32	1.04 ± 0.33	0.87 ± 0.24	0.88 ± 0.14	1.31 ± 0.06	0.80 ± 0.08	1.09 ± 0.01	1.08 ± 0.09
	60 µM	1.12 ± 0.09	1.26 ± 0.15	0.84 ± 0.20	1.01 ± 0.04	1.07 ± 0.26	1.25 ± 0.16	0.89 ± 0.02	1.00 ± 0.01	0.90 ± 0.20	1.06 ± 0.27
Topiramate (TOPI)	15 µM	1.51 ± 0.16 *	1.05 ± 0.14	0.97 ± 0.11	0.97 ± 0.09	1.02 ± 0.13	0.94 ± 0.07	1.40 ± 0.13	1.24 ± 0.23	1.23 ± 0.01	1.69 ± 0.33
	60 µM	1.28 ± 0.06	0.89 ± 0.14	1.06 ± 0.01	1.45 ± 0.09 *	1.27 ± 0.26	1.55 ± 0.08 *	1.59 ± 0.17 *	1.41 ± 0.03	1.64 ± 0.15 *	2.14 ± 0.24 *
Levetiracetam (LEVI)	40 µM	0.90 ± 0.13	0.75 ± 0.11	0.90 ± 0.10	1.02 ± 0.28	0.87 ± 0.11	0.78 ± 0.11	1.00 ± 0.05	1.06 ± 0.15	1.13 ± 0.19	0.96 ± 0.15
	120 µM	1.10 ± 0.16	0.95 ± 0.20	1.07 ± 0.14	1.07 ± 0.33	1.24 ± 0.01	0.72 ± 0.09	0.93 ± 0.04	0.87 ± 0.08	1.08 ± 0.21	1.13 ± 0.04

Real−time RT−PCR analysis of total mRNA isolated from Caco2 or HepG2 cells treated with PHT, CBZ, VAL, LAMO, TOPI and LEVI for 24 h. The changes in mRNA levels of target genes were normalized with β-microgloublin/GAPDH and expressed as normalized fold change over VC (vehicle control, 0.1% DMSO). The data are the means ± S.D. of at least (*n* = 5) independent real time PCR results. Statistical significance was determined using ANOVA. * indicates *p* < 0.05, VC (vehicle control, 0.1% DMSO) vs. PHT or VC vs. CBZ or VC vs. VAL or VC or TOPI. Grey window indicates significant change observed and focused at protein expression and functional activity of ABCB1 on treatment with CBZ and VAL whereas of ABCC2 on treatment with CBZ as shown in Figure 1. No significant change observed with other transporters on treatment with AEDs (protein data not shown).

**Table 2:** Sequence of primers used for Real time RT-PCR.

Primers		Sequence
ABCB1	Forward	GCCTGGCAGCTGGAAGACAAATAC
	Reverse	ATGGCCAAAATCACAAGGGTTAGC
ABCC1	Forward	TGTGTGGGCAACTGCATCG
	Reverse	GTTGGTTTCCATTTCAGATGACATCCG
ABCC2	Forward	ATATAAGAAGGCATTGACCC
	Reverse	ATCTGTAGAACACTTGACC
ABCG2	Forward	GAAGAGTGGCTTTCTACCTT
	Reverse	GTCCCAGGATGGCGTTGA
CYP3A4	Forward	TTGGAAGTGGACCCAGAAAC
	Reverse	CTGGTGTTCTCAGGCACAGA
B2M	Forward	GGCATTCCTGAAGCTGACAG
	Reverse	TGGATGACGTGAGTAAACCTG
GAPDH	Forward	ACATCGCTCAGACACCATG
	Reverse	TGTAGTTGAGGTCAATGAAGGG

## Supplementary Materials

Supplementary Materials are available online. Figure S1: Cell viability test using MTT assay, Figure S2: List of AEDs under study, structure, mode of action, discovery and marketed details along with their enzyme inducing nature, Figure S3: Strategy followed to understand antiepileptic drugs (AEDs)-mediated regulation of expression and function of ABC transporters.
