# Supplementary material for: In Vitro Assessment of the Effect of Antiepileptic Drugs on Expression and Function of ABC Transporters and Their Interactions with ABCC2
Source: Molecules. 2017 Sep 29;22(10):1484. doi: 10.3390/molecules22101484 (PMC6151573; doi:10.3390/molecules22101484)
Supplement: Supplementary file 1 [file molecules-22-01484-s001.pdf]

In vitro assessment of effect of antiepileptic drugs on expression and function of ABC transporters and their interactions with ABCC2

Gurpreet Kaur Grewal , Samiksha Kukal , Neha Kanojia, Krateeka Madan , Luciano Saso and Ritushree Kukreti

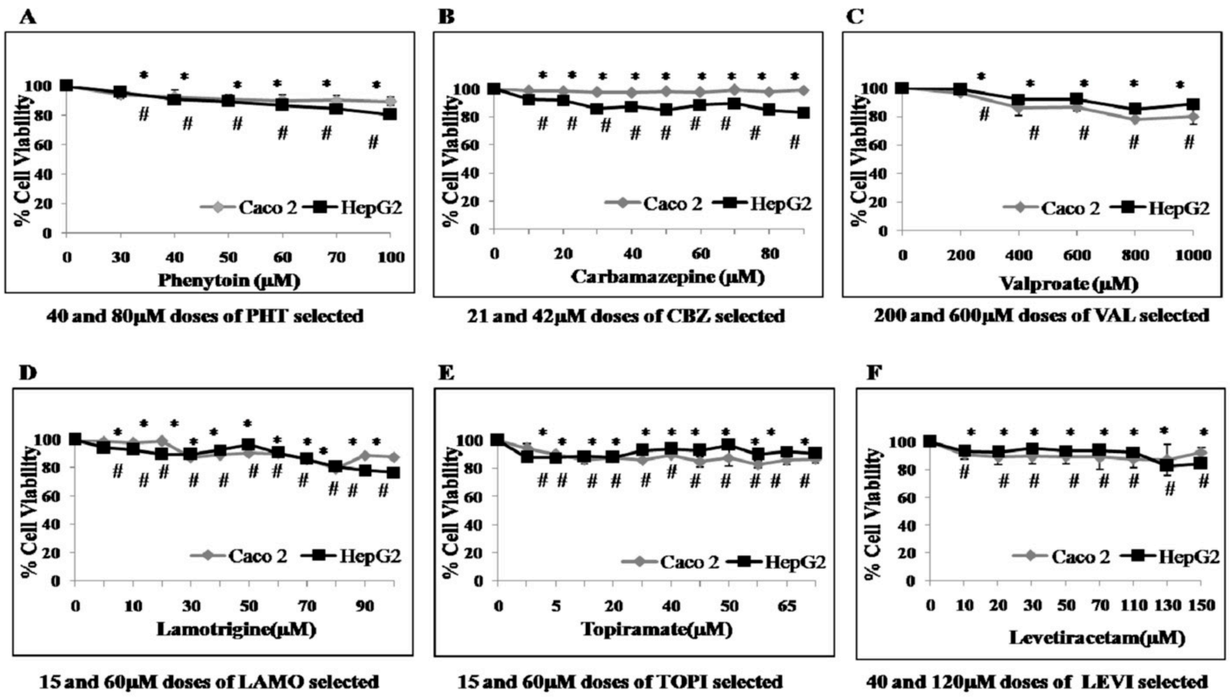

Figure S1. Cell viability test using MTT assay.

MTT assay was performed with (A) Phenytoin, (B) Carbamazepine, (C) Valproic acid, (D) Lamotrigine, (E) Topiramate and (F) Levetiracetam in HepG2 cells. Cells (10 000 cells/well-HepG2) were plated in 96-well plates for 24 h and subsequently treated with varying concentration of drugs for 72 h. Data represents the mean ± S.D. of five (n = 5) independent experiments. Statistical significance (*, $p < 0.05$ for HepG2) (#, $p < 0.05$ for Caco2) was determined using ANOVA followed by Tukey's HSD post hoc test. Non-cytotoxic and therapeutic doses were used for further experiments in the study. For the first line AEDs namely PHT, CBZ and VAL doses selected were 40 and 80µM, 21 and 42µM and 200 and 600µM respectively. For the second line AEDs namely LAMO, TOPI and LEVI doses selected were 15 and 60µM, 15 and 60µM and 40 and 120µM respectively.

AED	Structure	Mode of action	Discovery	Marketed	Enzyme Inducer
Phenytoin	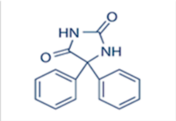	Sodium channel blocker	1908	1938	CYP3A4
Carbamazepine	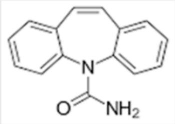	Sodium channel blocker	1953	1962	CYP3A4
Valproate	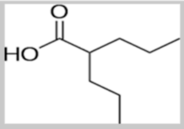	Sodium channel blocker	1881	1978	CYP3A4
Lamotrigine	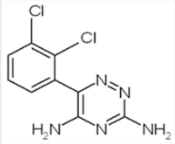	Sodium channel blocker ; GABA action	-	1994	-
Topiramate	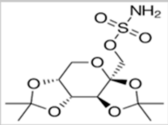	Glutamate blocker	1979	1997	CYP3A4
Levetiracetam	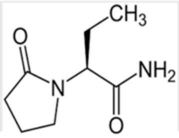	Calcium channel blocker	-	1999	-

Figure S2. List of AEDs under study, structure, mode of action, discovery and marketed details along with their enzyme inducing nature.

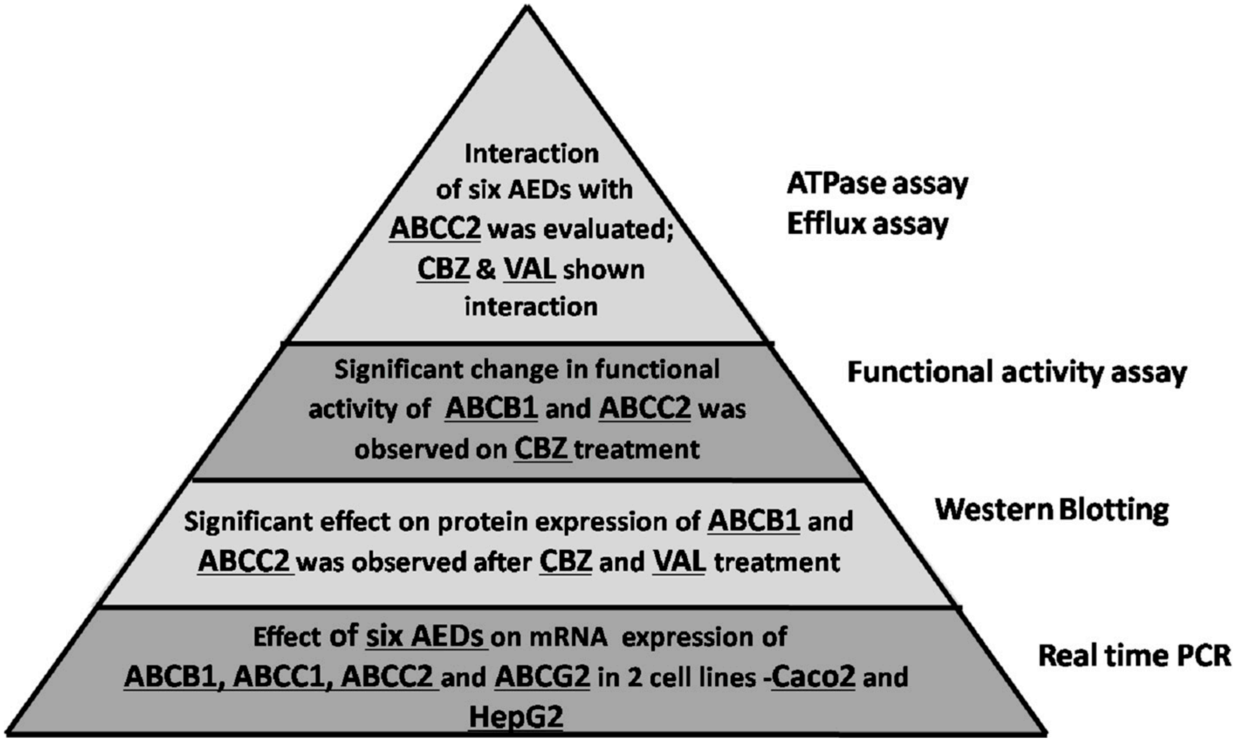

Figure S3. Strategy followed to understand antiepileptic drugs (AEDs)-mediated regulation of expression and function of ABC transporters
